# Urban blue–green space landscape ecological health assessment based on the integration of pattern, process, function and sustainability

**DOI:** 10.1038/s41598-022-11960-9

**Published:** 2022-05-11

**Authors:** Shuang Song, Shaohan Wang, Mengxi Shi, Shanshan Hu, Dawei Xu

**Affiliations:** 1 College of Landscape Architecture, Northeast Forestry University, Harbin, 150000 China; 2 Key Lab for Garden Plant Germplasm Development and Landscape Eco-Restoration in Cold Regions of Heilongjiang Province, Harbin, 150000 China

**Keywords:** Ecological modelling, Urban ecology, Wetlands ecology, Drug discovery

## Abstract

Landscape ecological health (LEH) assessment of blue–green space is vital for the management and restoration of the urban environment. At present, existing LEH assessment research has mainly focused on the single measurement of landscape pattern or external ecological service function, ignoring the effect mechanism. Moreover, there is a lack of targeted assessment of urban blue–green space LEH. In this study, we constructed an urban blue–green space LEH assessment framework based on the integration of pattern, process, function and sustainability, and conducted an empirical analysis in Harbin, a megacity in Northeastern China. The results showed that the spatial changes in the four assessment units of landscape ecological pattern, process, function and sustainability were not coordinated in the study area. From 2011 to 2020, the overall condition of blue–green space LEH in the study area improved but still at an unhealthy level, and the spatial difference increased. Grassland, water and wetland suffered from the widespread degradation of LEH in the study area, and the LEH level improvement type had the largest area proportion, and the stabilization type had the smallest. Moreover, based on the spatial autocorrelation analysis, we clarified the LEH spatial correlation characteristics of the study area and proposed targeted optimization suggestions. Our assessment framework will extend the LEH assessment scope and methodology, and the research results can provide significant references for urban blue–green space protection and management.

## Introduction

Landscape ecological health (LEH) is a new and intersecting ecology concept in the field of landscape ecology and ecosystem health. LEH mainly studies ecosystem issues that are severely affected or degraded or even disappeared under intense human disturbance^1–3^. The spatial scale of the landscape is between the ecosystem and the region, and it is the broad spatial scale most closely related to human beings^4,5^. At such a spatial scale, LEH is more suitable for conducting targeted research on resource and environmental issues^6–8^. Meanwhile, the natural regulation process at the landscape scale has high stability, which can better reflect the development rule of ecosystems^9,10^. Urban blue–green space is the most severely disturbed ecosystem by human social and economic activities^5,11^, its status is directly related to the sustainable development of the urban environment. Therefore, it is of great significance to study the ecological health of urban blue–green space at the landscape scale.

To date, the LEH research has roughly undergone a transformation from macro-discussion to micro-survey, from overall trend structure to specific driving factor analysis^12,13^. The early research achievements are mainly concept elaboration, research progress, and results review^14,15^. These achievements have laid the foundation for promoting theory research, but have limited guidance for solving specific problems. After that, the research on LEH has mainly focused on the quality of landscape ecology planning, the balance between ecological health and landscape aesthetics, the construction of a comprehensive ecological index system, the spatial patterns and evolutionary characteristics, and so on^15–17^. And the LEH study scale is mainly in watershed or municipality, the objects mainly focused on forests, wetlands, islands, and parks^18,19^.

The urban blue–green space is driven by both human activities and natural disasters, resulting in changes in landscape pattern, process, and function that ultimately lead to LEH deterioration^20,21^. The concept of blue–green space was first proposed in 2015, but there is no specific explanation of the definition^20–22^, and researchers generally understand blue–green space as a composite space made up of blue space represented by rivers and lakes and green space represented by grasslands. As a semi-natural ecosystem, there is an argument about whether cultivated land belongs to blue–green space. Taylor & Hochuli (2017) proposed two definitions of urban blue–green space at macro and micro levels after analyzing and summarizing 125 journal articles on urban blue–green space^23,24^. The macro-level refers broadly to ecological areas in general, including water bodies and vegetated areas, such as forests, coastal areas, cultivated areas, parks, and gardens. The micro-level refers to the open space covered by vegetation, including urban parks, gardens, courtyards, urban forests, and urban farms. As a link between various natural patches, cultivated land has an irreplaceable role in the stable development of urban ecology, promoting biodiversity, and improving the comprehensive ecological health of the city. It interlaces with other types of ecological patches, sharing risks and complementing each other to form the urban landscape ecological pattern^25^. Meanwhile, these patches together embody the qualities and potentials of recreation, economy, and ecology to maintain the ecological balance and development of the urban landscape. Thus, cultivated land is an important part of the blue–green space.

The existing assessment studies on urban blue–green space LEH are rare and unsystematic, and the critical to scientifically carrying out the urban blue–green space LEH assessment is the selection of methods. Common methods include the indicator species method and the index system method. The indicator species method is unclear in terms of selection criteria and the indicated extent for LEH^5,9^. And it cannot take into account many factors such as economic, social, and human health, which makes it difficult to reflect LEH in an accurate and scientific way^17^. The index system method could cover a wide range of factors besides ecology, so its results are more comprehensive. At present, a series of assessment models are available, such as Ecological-Feature-Function-Social-Economic Model, Drive-Pressure-State-Exposure-Effect-Response (DPSEEA) model, Pressure-State-Response (PSR) model, Vigor-Organization-Resilience (VOR) model, Condition-Vigor-Organization-Resilience (CVOR) model and so on^9,17^. These models could assess the status of the object fairly well, but the research on the nature and mechanism of LEH is insufficient, and practical guidance is limited. Urban planning, ecological safety, and social development all rely on blue–green space, which is an irreplaceable ecological carrier. It is urgent to build a scientific and comprehensive LEH assessment framework of urban blue–green space.

**Figure 1:**
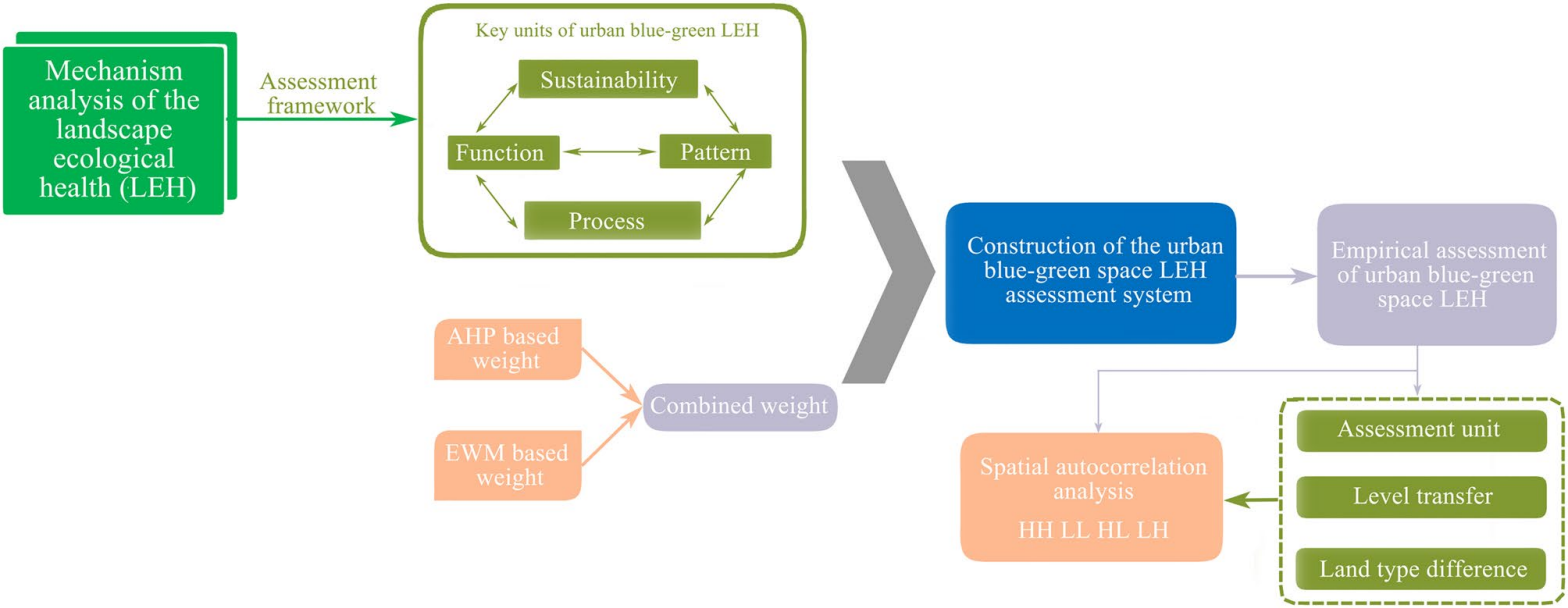
The flowchart of present research.

Harbin is an important hub for the first Eurasian land bridge and air corridor, a significant city for development and opening along the border in China^22–24^. With the introduction of “Measures of the State Council on Revitalizing the Old Industrial Bases of Northeast China” in 2009, the further acceleration of resource-based cities transfer has posed higher challenges to the development and management of blue–green space in Harbin^25,26^. Thus, it is significant to identify the LEH status and spatial and temporal change characteristics. The objectives of this study were to (1) construct a scientific and integrated urban blue–green space LEH assessment framework by clarifying the effect mechanism; (2) conduct an empirical assessment of blue–green space in the study area from 2011 to 2020; (3) identify the spatial and temporal changes of blue–green space LEH in the study area; (4) clarify the LEH spatial correlation characteristics based on the spatial autocorrelation analysis. The study results will provide theoretical and practical support for the formulation of a rational and scientific urban blue–green space development strategy during the rapid urban expansion. Figure 1 presents the flowchart of present research.

## Materials and methods

### Study area

**Figure 2:**
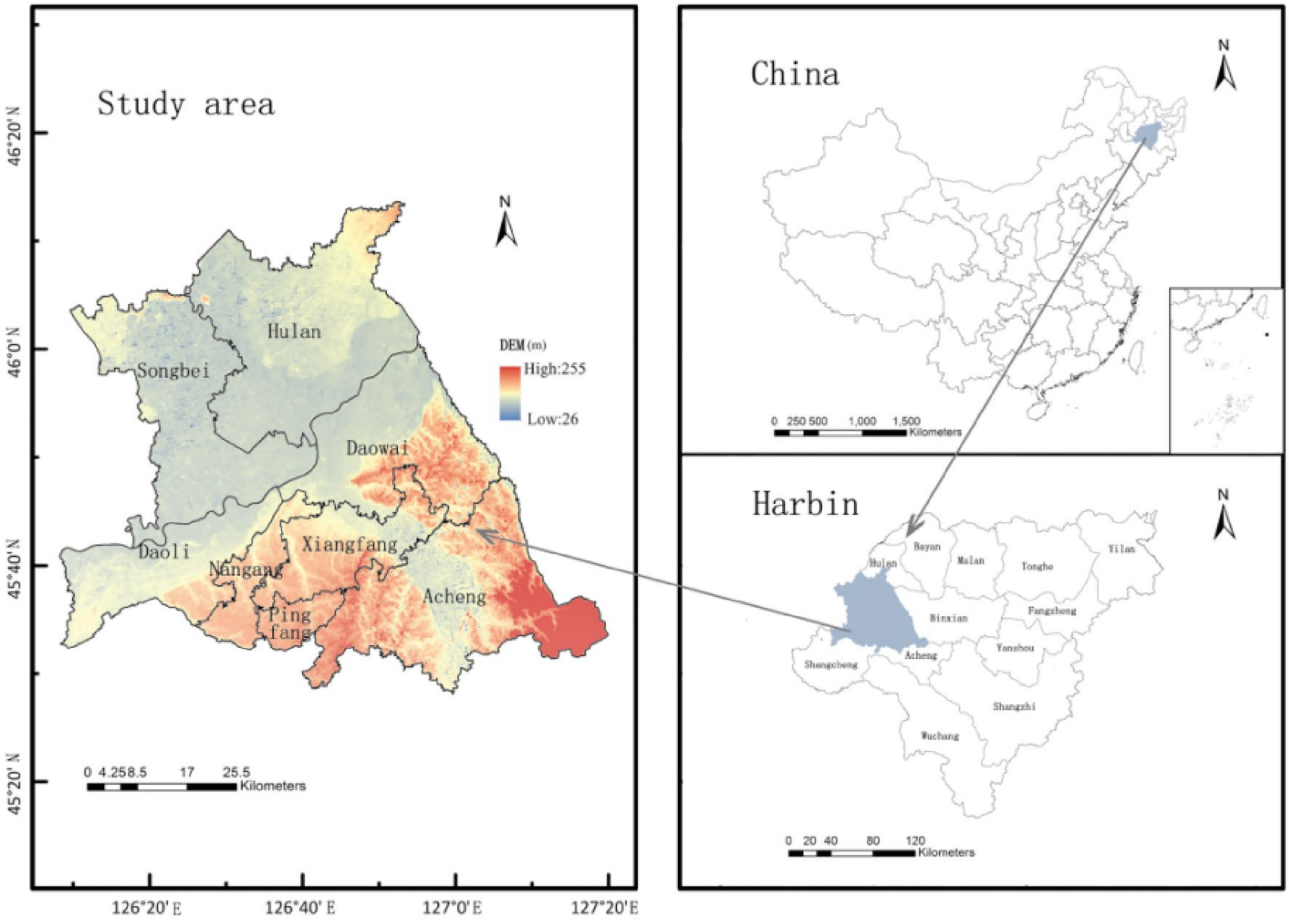
Schematic of study area. The Figure is created using ArcGIS ver.10.2 (https://www.esri.com/).

Harbin is located in the centre of Northeast Asia, between 44°04'–46° 40′ N and 125° 42′–130° 10′ E^24,26^. The site has a mid-temperate continental monsoon climate, with an average annual temperature of 3.6° C and an average annual precipitation is 569.1 mm. The main precipitation months being from June to September, accounting for about 60% of the annual precipitation, the main snow months are from November to January^24,25^. The overall topography is high in the east and low in the west, with mountains and hills predominating in the east and plains predominating in the west^27^. In this study, we identified the central district of Harbin, where urban construction activities are frequent and the population is dense, as the study area. According to the “Harbin City Urban Master Plan (2011–2020)” (revised draft in 2017), the specific scope includes Daoli District, Daowai District, Nangang District, Xiangfang District, Pingfang District, Songbei District's administrative district, Hulan District, and Acheng District part of the area, with a total area of 4187 km^2^ (Fig. 2). The blue–green space in this study included woodland, grassland, cultivated land, wetland and water that permeate inside and outside the construction sites. They all have integrated functions such as ecology, supply, beautification, culture, and disaster prevention and avoidance, and have a decisive influence on the urban ecological environment.

### Data sources

The data used in this research included the following: land-cover date (30 m × 30 m) of two periods (2011, 2020) spported by the China Geographic National Conditions Data Cloud Platform (http://www.dsac.cn/), Meteorological datasets (1 km × 1 km) were obtained from the Resource and Environmental Science Data Center of the Chinese Academy of Sciences (http:∥www.resdc.cn/), including air temperature, precipitation, and surface runoff. ASTER GDFM elevation data (30 m × 30 m) came from the Geospatial Data Cloud (http:∥www.gscloud.cn), from which the slope was extracted. Soil data (1 km × 1 km) were from the World Soil Database (HWSD) China Soil Data Set (v1.1). The normalized difference vegetation index (NDVI) and modified normalized difference water index (MNDWI) data (30 m × 30 m) came from the National Comprehensive Earth Observation Data Sharing Platform (http://www.chinageoss.org/), ET datasets (30 m × 30 m) were drawn from the NASA-USGS (https://lpdaac.usgs.gov/). Social and economic data were mainly obtained through the Harbin statistical yearbook and the Harbin social and economic bulletin.

### Framework of urban blue–green space LEH assessment

Urban blue–green space is a politically defined man-land coupling region composed of ecological, economic, and social systems, which is greatly disturbed by human activities^11^. The essence of urban blue–green space LEH is that the landscape ecological function sustainably meets human needs^28,29^. The landscape ecological function reflects the value orientation of human beings to blue–green space, and to a large extent affects the blue–green landscape ecological pattern and process. The interaction between the blue–green landscape ecological pattern and process drives the overall dynamics of blue–green space. Meanwhile, presenting certain landscape ecological function characteristics, which provide ecological support for various human activities^30–32^. While the pattern and process of blue–green space both profoundly influence and are influenced by human activities^33,34^. This influence is long-term, the standard of LEH should not be fixed in real-time health, but should fully consider the sustainability of the health state.

**Figure 3:**
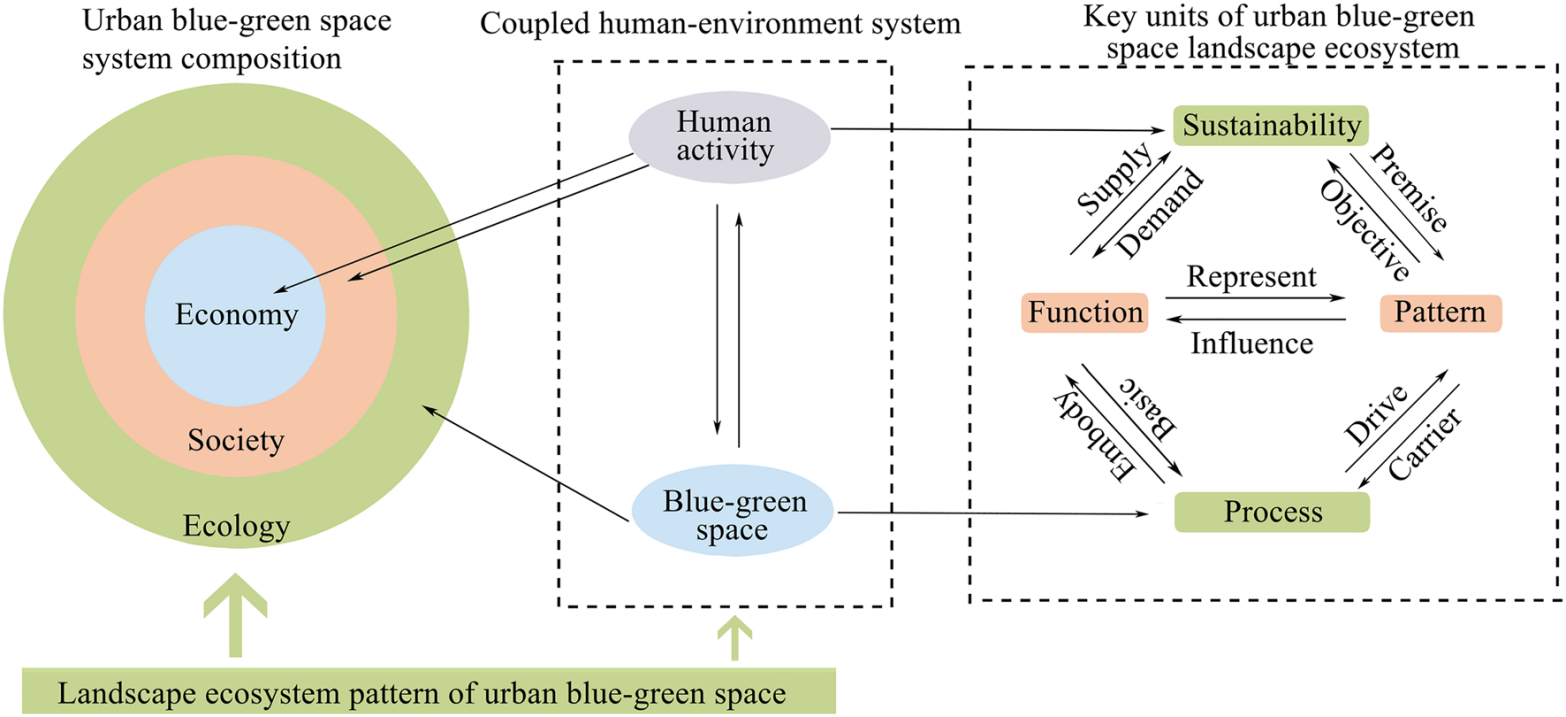
Key units, interactions of urban blue–green space LEH.

In summary, the landscape ecological pattern, process, function, and sustainability are not separate, but a complex of mutual integration, and organic unity. In this study, we constructed an integrated assessment framework of blue–green space LEH that included four units: pattern, process, service, and sustainability (Fig. 3). In the assessment framework, the LEH of urban blue–green space involves two dimensions: the first is the health status of the urban blue–green space itself, emphasizing the maintenance of the ecological conditions, thereby potentially satisfying a series of diversity goals. The other is that urban blue–green space, as a part of social and economic development, could sustainably provide the ability to meet (subject) needs and goals.

#### Landscape ecological pattern

The landscape ecological pattern of urban blue–green space is a spatial mosaic combination of landscape elements at different levels or the same level. Affected by human activities interference^31^, the landscape ecological pattern shows the changing trend of landscape structure complexity, landscape type diversification, and landscape fragmentation. The assessment of urban landscape ecological pattern should be a comprehensive reflection of this changing trend^1^. Landscape pattern indexes are the most frequently applied which could reflect the structural composition and spatial configuration characteristics of the landscape^4,35^. This study took landscape ecology as the entry point and selected the landscape pattern indexes that can quantitatively reflect the change characteristics of landscape structural composition and spatial configuration under the disturbance. In this way, the landscape disturbance index (U), landscape connectivity index (CON), and landscape adaptability index (LAI) were used as the indexes for the assessment of landscape ecological pattern health.

1. Landscape disturbance index (U)

There are two kinds of relationships between the landscape ecological pattern and the external disturbance: compatibility and conflict. As the landscape ecological pattern has accommodating characteristics, the disturbance beyond the accommodating capacity will degrade the landscape ecological pattern^36,37^. The landscape disturbance index (U) could characterize the degree of fragmentation, dispersion, and morphological changes in landscape pattern^38^. The index is a comprehensive index that can reflect the health of the landscape pattern by quantifying the ability of ecosystems to accommodate external disturbances. It consists of the landscape fragmentation index, the inverse of the fractional dimension, and the dominance index. They measure the response of the landscape pattern to external disturbance from the perspective of different landscape types, the same landscape type, and landscape diversity, respectively^36,38^, and their weights were determined by the entropy weight method. The formula is as follows:

1 $$ U = \alpha N_{{{Fi}}} + bD_{{{Fi}}} + cD_{{{Oi}}} $$

where *N*_Fi_ is the landscape fragmentation index, *D*_Fi_ is the inverse of the fractional dimension, *D*_Oi_ is the dominance index, and *a*, *b*, and *c* are the corresponding weights, which were 0.20, 0.5, and 0.3 in this study, respectively.

(2) Landscape connectivity index (CON)

The most direct result of landscape ecological pattern degradation caused by external disturbance is that the flow of energy, material, and information among ecological patches is reduced or even blocked, ultimately the stability of the landscape pattern is decreased. The connectivity could characterize the ability of landscape ecological pattern to mitigate risk transmission, which is significant for the dynamic stability of landscape ecological pattern^39,40^. The landscape connectivity index (CON) could measure the connectivity between ecosystem components through the aggregation or dispersion trend of patches^41^. The better the connectivity, the stronger the stability of landscape ecological pattern. The formula is as follows:

2 $$ CON = \frac{{100\sum\limits_{s = 1}^{q} {\sum\limits_{h \ne l}^{p} {C_{{{shl}}} } } }}{{\sum\limits_{s = 1}^{s} {\left[ {q_{{s}} (q_{{s}} - 1)/2} \right]} }} $$

where *q*_s_ is the number of plaques of patch type *s*, *C*_shl_ is the link between patch *h* and patch *l* in *s* within the delimited distance.

(3) Landscape Restorability Index (LRI)

The ability to recover to its original structure when subjected to disturbances is an important criterion for the landscape ecological pattern^42^. Research confirmed that the restorability of the landscape ecological pattern is closely related to the structure, function, diversity, and uniformity of distribution. The landscape restorability index (LRI) combines the above landscape information and could indicate the restorability of the landscape ecological pattern in response to disturbance^43^. The index consists of the patch density, Shannon diversity index, and the landscape evenness, the patch density is the number of patches per square kilometer. The Shannon diversity index reflects the change in the proportion of landscape types. The landscape evenness index shows the distribution evenness of patches in terms of area. The larger the LRI index, the more complex and evenly distributed the structure is, and the more recovery ability of the landscape pattern against disturbance is. The formula is as follows:

3 $$ LRI = PD \times SHDI \times SHEI $$

where *PD* is the patch density, *SHDI* is the Shannon diversity index, and *SHEI* is the landscape evenness index.

#### Landscape ecological process

The landscape ecological process of urban blue–green space is extremely complex for it involves multiple factors such as natural ecology, economy, and culture. Landscape ecological process assessment is the measure of the self-organized capacity and the efficiency of ecological processes within and among patches^44^. A blue–green space with a healthy landscape ecological process should have the ability to adapt to conventional land use under human management and maintain physiological integrity while maintaining the balance of ecological components. Specifically, the landscape ecological process could quickly restore its balance after severe disturbances, with strong organization, suitability, recoverability, and low sensitivity^45,46^. A single model hardly to gets good research on landscape ecological process under the urban scale. The comprehensive application of multidisciplinary methods is effective means to solve the problem. Regarding this, we selected ecological indexes and models from four aspects: organization, suitability, restoration, and sensitivity to assess the landscape ecological process of urban blue–green space.

1. Organization index (O)

The organization of the landscape ecological process is the maintenance ability of stable and orderly material cycling and energy flow within and between landscapes^47^. The normalized vegetation index (NDVI) and the modified normalized difference water index (MNDWI) could reflect the efficiency and order of ecological processes. Such as accumulation of organic matter, fixation of solar energy, nutrient cycling, regeneration, and metabolism^13^. The indexes are the external performance of the internal dynamics and organizational capabilities of the ecological process. In recent years, it has been widely used in the assessment of related to landscape ecological process. The formulas are as follows:

$$ NDVI = \frac{NIR - R}{{NIR + R}} $$

4 $$ MNDWI = \frac{p(green) - p(MIR)}{{p(green) + p(MIR)}} $$

where $NDVI$ is the normalized vegetation index, $MNDWI$ is the modified water body index, $NIR$ is the reflectance value in the near-infrared band, $R$ is the reflectance value in the visible channel, $p(green)$ and $p(MIR)$ are the normalized values in the green and mid-infrared bands.

(2) Suitability index (Q)

The suitability of the landscape ecological process is a measurement of the self-regulating ability of the landscape ecosystem. That is, to effectively maintain the ecological process in a state of being protected from disturbance during the occasional changes caused by the external environment^2^. The water conservation amount index (Q) can measure the operating capacity of ecosystems to maintain ecological balance, water conservation, climate regulation, and other ecological processes by integrating the water balance of rainfall, surface runoff, and evaporation^41^. It could reflect the suitability of landscape ecological process to regional environment and developmental conditions. The formula is as follows:

5 $$ Q = R - J - ET $$

where *Q* is the water conservation amount, *R* is the annual rainfall, *J* is the surface runoff, *ET* is the evapotranspiration.

(3) Recoverability index (ECO)

The recoverability of the landscape ecological process refers to the ability of an ecosystem to return to its original operating state after being subjected to external impacts. Land-use types play an essential role in landscape ecological recoverability^48^. The ecological recoverability index (ECO) uses the resilience coefficients of land-use types to reflect the level of ecosystem resilience^38^. Based on previous studies, the resilience coefficient of land-use types was assigned (Table 1).

(4) Sensitivity index(A)

**Table 1:** Resilience coefficients of different land use types.

Land-use types	Cultivated land	Woodland	Grassland	Water area	Wetland	Construction land	Unused land
Resilience coefficients	0.3	0.8	1.0	0.8	0.7	0.4	0

The sensitivity index (A) could be used to indicate landscape ecological process formation, change, and vulnerability to disturbance^31^. We started from the physical effects of blue–green space on sand production, water confluence, and sediment transport, introduced the Soil Erosion Modulus to characterize the sensitivity of landscape ecological processes to disturbance. The index effectively combines landscape ecology, erosion mechanics, soil science, and sediment dynamics^49^. The formula is as follows:

6 $$ \begin{gathered} A = R_{{i}} \cdot K \cdot LS \cdot C \cdot P \hfill \\ L = (l/22.1)^{m} \hfill \\ S = \left\{ \begin{gathered} 10.8\sin \theta + 0.03,\theta < 5^{ \circ } \hfill \\ 16.8\sin \theta - 0.50,5^{ \circ } \le \theta < 10^{ \circ } \hfill \\ 21.9\sin \theta - 0.96,\theta \ge 10^{ \circ } \hfill \\ \end{gathered} \right. \hfill \\ C = \left\{ \begin{gathered} 1,c = 0 \hfill \\ 0.6508 - 0.3436\lg c,0 < c \le 78.3\% \hfill \\ 0,c > 78.3\% \hfill \\ \end{gathered} \right. \hfill \\ \end{gathered} $$

where *A* is the soil erosion modulus. *R*_i_ is the rainfall erosion factor, *K* is the soil erosion factor, *L* and *S* are slope the length factor and the slope factor respectively, *C* is the vegetation coverage and management factor, *P* is the soil and water conservation factor, *l* is the slope length value, *m* is the slope length index, and *θ* the is slope value.

#### Landscape ecological function

The landscape ecological function determines the ability of ecological service^50–52^, the ecological service of urban blue–green space depends on the human value orientation^48^. It includes four categories: supply, support, regulation, and culture. Based on Maslow's Hierarchy of Needs and Alderfer’s ERG theory, scholars have summarized the three major needs of human beings for urban blue–green space. Namely, securing the living environment to meet the survival needs, improving social relationships to meet the interaction needs, and cultivating cultural cultivation to meet the development needs^53^. Specifically corresponding to the landscape ecological function of urban blue–green space, supply is not the main function, only plays a subsidiary role, support is the basic guarantee, regulation is the basic need for urban environmental construction, and culture is an important element of high-quality social life. Ecosystem service value (ESV) can realize the measurement of ecological service function by calculating the specific value of life support products and services produced by the ecosystem^54–56^. Considering the human value orientation of the urban blue–green space landscape ecological function, the weights were given by consulting 16 experts, with supply, regulation, support, and culture weights of 0.2, 0.3, 0.3, 0.2, respectively. The formula is as follows:

7 $$ ESV = \sum\limits_{k = 1}^{n} {S_{k} \times V_{k}^{{}} } $$

where *S*_k_ is the area of landscape type *k*, *V*_k_ is the value coefficient of the ecosystem service function of landscape type *k .*

#### Landscape ecological sustainability

Wu (2013) proposed a research framework for landscape sustainability based on a summary of related studies, stating that landscape ecological sustainability is the ability to provide ecosystem services in a long-term and stable manner^34^. The framework emphasized that landscape sustainability should focus on the analysis of ecosystem service trade-offs effect^34,57^. In the process of dynamic change of urban blue–green space ecosystem, there are complex trade-offs among various ecosystem services. This is important for promoting the optimal overall benefits of various ecosystem services and achieving sustainable development of urban ecology^58^. In addition, as a special type of human-centered ecosystem developed by humans based on nature, human well-being is also very important for the landscape ecological sustainability of urban blue–green space. For this reason, we introduced ecosystem service trade-offs (EST) and ecological construction input (IEC) as assessment indexes of landscape ecological sustainability.

1. Ecosystem service trade-offs (EST)

This study applied the root mean square deviation of ecological services to quantify ecosystem service trade-offs (EST). The index could effectively measure the average difference in standard deviation between individual ecosystem services and the average ecosystem services. It is a simple and effective way to evaluate the trade-offs among ecosystem services. The formula is as follows:

8 $$ EST = \sqrt {\frac{1}{n - 1}\sum\nolimits_{i = 1}^{n} {(ES_{std} - \overline{ES}_{std} } } )^{2} $$

where *ES*_*std*_ is the normalized ecosystem services, n is the number of ecosystem services , and $\overline{ES}_{std}$ is the mean value of normalized ecosystem services.

(2) Ecological construction input (ECI)

Human well-being is a premise for the landscape ecological sustainability of urban blue–green spaces, it is closely related to government investment in ecological construction planning^34^. From the perspective of economics, this study assessed the human well-being obtained by urban blue–green space with the ratio of urban ecological construction investment to GDP, that is, the ecological construction input (ECI). The formula is as follows:

9 $$ ECI = EI/G $$

where *EI* is the amount of ecological construction investment, and *G* is the gross regional product.

### Evaluation method

The index weight determines its relative importance in the index system, and the selection of the weight calculation method in the decision-making of multi-attribute problems has an important impact on the assessment results^21^. Traditional weighting methods can be divided into two categories, subjective weighting method and objective weighting method^21,38^. The subjective weighting method is represented by the analytic hierarchy process (AHP), Delphi method, and so on. It has the advantage of simplicity, but the disadvantage is too subjective and randomness because it was completely dependent on the knowledge and experience of decision makers. The objective weighting method is represented by the entropy weighting method (EWM), principal component analysis, variation coefficient method, and so on. And it has been widely recognized for reflecting the variability of assessment results^18^, but the values of indexes have significant influence and the calculation results are not stable. Considering the limitations of the single weighting method, the weights of each assessment index in this study were determined by the combination of subjective weight and objective weight. Among them, the subjective weighting selected the AHP, and the objective weighting selected the EWM (Table 2). The formula is as follows:

10 $$ w_{{j}} = \alpha w_{{j}}^{{{AHP}}} + (1 - \alpha )w_{{j}}^{{{EWM}}} $$

11 $$ w_{{j}}^{{{EWM}}} = d_{{j}} /\sum\limits_{i = 1}^{m} {d_{{j}} } $$

12 $$ d_{{j}} = 1 - e_{{j}} $$

13 $$ e_{{j}} = - k\sum\limits_{i = 1}^{n} {f_{{{ij}}} \ln (f_{{{ij}}} )} ,\;k = 1/\ln (n) $$

14 $$ f_{{{ij}}} = X^{\prime}_{{{ij}}} /\sum\limits_{i = 1}^{n} {X^{\prime}_{{{ij}}} } $$

where $W_{{j}}^{{}}$ is the combined weight. $W_{{j}}^{{_{AHP} }}$ is the weight of the j-th index of the AHP, $W_{{j}}^{{{EWM}}}$ is the weight of the j-th index of the EWM, d_j_ is the information entropy of the j-th index, e_j_ is the entropy value of the j-th index, $f_{{{ij}}}$ is the proportion of the index value of the j-th sample under the i-th indexm, $X^{\prime}_{{{ij}}}$ is the standardized value of the i-th sample of the j-th index, m is the number of index, n is the number of samples, and $\alpha$ was taken as 0.5.

**Table 2:** Weight of assessment index.

Index	$$W_{{j}}^{{{EWM}}}$$$$( \pm )$$	$$W_{{j}}^{{{AHP}}}$$$$( \pm )$$	$$W_{{j}}$$$$( \pm )$$
Landscape disturbance	0.070 (−)	0.078 (−)	0.074 (−)
Landscape connectivity	0.085 ( +)	0.088 ( +)	0.086 ( +)
Landscape restorability	0.093 ( +)	0.082 ( +)	0.088 ( +)
Organization index	0.093 ( +)	0.111 ( +)	0.102 ( +)
Suitability index	0.096 ( +)	0.110 ( +)	0.103 ( +)
Recoverability index	0.106 ( +)	0.102 ( +)	0.104 ( +)
Sensitivity index	0.090 (−)	0.094 (−)	0.092 (−)
Ecological service value	0.192 ( +)	0.168 ( +)	0.180 ( +)
Ecosystem service trade-offs	0.081 (−)	0.077 (−)	0.079 (−)
Environmental construction input	0.094 ( +)	0.090 ( +)	0.092 ( +)

( +) positive index, (−) negative index.

Since the dimensions of indexes are different, it is necessary to unify the dimensions of the index to avoid the errors caused by direct calculation to make the evaluation results inaccurate. The range standardization was used to normalize the index data and bound its value in the interval [0, 1], the range standardization can be expressed as follows^15,23^:

15 $$ {\text{Positive indicator}}\left( + \right):A_{{{ij}}} = (X_{{{ij}}} - X_{{{jmin}}} )/(X_{{{jmax}}} - X_{{{jmin}}} ) $$

16 $$ {\text{Negative indicator}}\left( - \right):A_{{{ij}}} = (X_{{{jmax}}} - X_{ij} )/(X_{{{jmax}}} - X_{{{jmin}}} ) $$

Additionally, we divided the LEH index into five levels from high to low using an equal-interval approach as follows^40^: [1–0.8) healthy, [0.8–0.6) sub-healthy, [0.6–0.4) moderately healthy, [0.4–0.2) unhealthy, [0.2–0] pathological, corresponding level I–V. And the level transfer of LEH in different periods was divided into three types: improvement type, degradation type, and stabilization type. For example, III-II means that the transfer from level III to level II is the improvement type.

### Spatial autocorrelation analysis

Spatial autocorrelation analysis is one of the basic methods in theoretical geography. It could deeply investigate the spatial correlation characteristics of data, including global spatial autocorrelation and local spatial autocorrelation^23^. The global spatial autocorrelation uses global Moran’s I to evaluate the degree of their spatial agglomeration or differentiation of an attribute value in the study area. The local spatial autocorrelation is a decomposed form of the global spatial autocorrelation^18,21^, including four types: HH(High-High), LL(Low-Low), HL(High-Low), LH(Low–High). In this study, spatial autocorrelation analysis was applied to study the spatial correlation characteristics of blue–green space LEH. The calculation formulas are as follows:

17 $$ I = \frac{{N\sum\limits_{i} {\sum\limits_{v} {W_{iv} (Y_{i} - \overline{Y} )(Y_{v} - \overline{Y} )} } }}{{(\sum\limits_{i} {\sum\limits_{v} {W_{iv} } } )\sum\limits_{i} {(Y_{i} - \overline{Y} )} }} $$

18 $$ I_{i} = \frac{{Y_{i} - \overline{Y} }}{{S_{x}^{2} }}\sum\limits_{v} {\left[ {W_{iv} (Y_{i} - \overline{Y} )} \right]} $$

where N is the number of space units, $W_{iv}$ is the spatial weight, $Y_{i} ,Y_{v}$ are the variable attribute values of the area $i,v$, $\overline{Y}$ is the variable mean, $S_{x}^{2}$ is the variance, $I$ is the global Moran’s I index, and $I_{i}$ is the local Moran’s I index.

## Results

### Assessment unit of urban blue–green space LEH

#### Landscape ecological pattern

**Figure 4:**
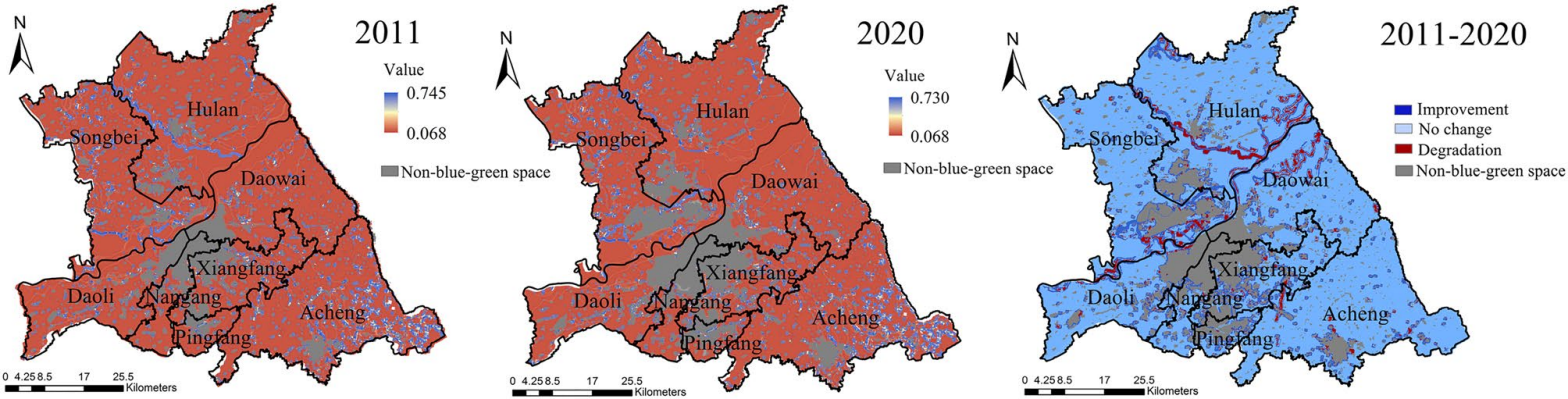
Spatial distribution of landscape ecological pattern. The Figure is created using ArcGIS ver.10.2 (https://www.esri.com/).

The landscape ecological pattern of the study area in 2011 and 2020 had the same maximum (0.730) value and mean (0.392) value, with an overall spatial distribution of higher west than east, higher north than south (Fig. 4). The high values of the landscape ecological pattern in both periods were mainly distributed in Acheng and Songbei Districts. The low values were mainly concentrated in Pingfang, Nangang, and Xiangfang Districts. Compared with 2011, the landscape ecological pattern in 2020 had a decrease in the minimum value and a larger mean square error. The improvement areas were mainly concentrated in the north of Daowai District, the west and south of Acheng District, the east of Songbei District, and the south of Nangang District. Although the blue–green spatial patches in these areas were mostly artificial, they formed a network of patches with good interactions^26^. The Degradation areas were gathered in the north of Hulan District, the central part of Acheng District, and the southern part of Songbei District. In these areas, the blue–green space patches were large scale and few, and the regional ecological network was weak and unformed.

#### Landscape ecological process

**Figure 5:**
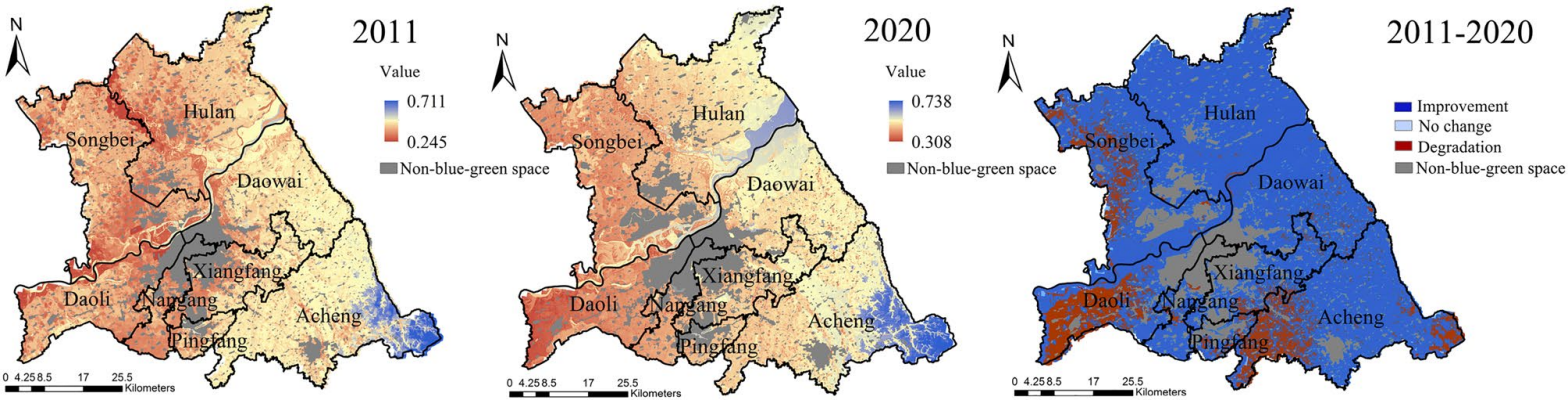
Spatial distribution of landscape ecological process. The Figure is created using ArcGIS ver.10.2 (https://www.esri.com/).

The overall distribution of landscape ecological process in the study area was high in the east and low in the west, high in the south and low in the north in both periods (Fig. 5). The high values of the landscape ecological process in 2011 emerged in the Acheng District, the north and east of Daowai District, and the east of Hulan District. The low values were concentrated in Xiangfang, Nangang, and Pingfang Districts. In 2020, the minimum, maximum, and mean values of the landscape ecological process were higher than those in 2011, and the overall trend was on the rise. Among them, the landscape ecological process had a certain degree of degradation in the north-central part of Songbei District, Daoli District, and the west of Acheng District.

#### Landscape ecological function

**Figure 6:**
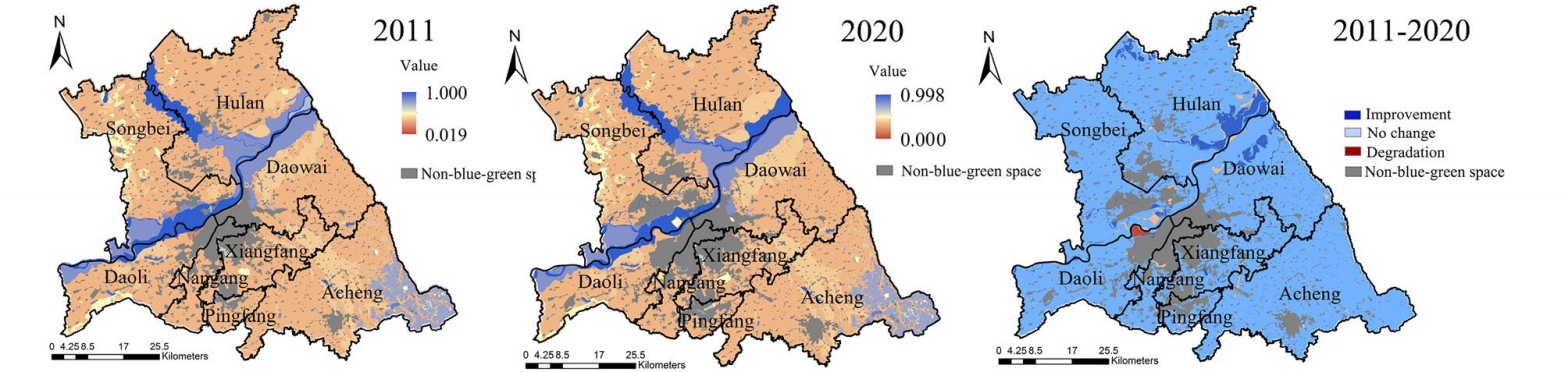
Spatial distribution of landscape ecological function. The Figure is created using ArcGIS ver.10.2 (https://www.esri.com/).

The high values of landscape ecological function in the study area were located in the water and wetland. The low values were concentrated in the beach in the southeast of Hulan District, the northwest of Daowai District, and the south of Daoli District. Due to the large proportion of dry land, the overall of landscape ecological function was at a low level in the study area (Fig. 6). In 2020, the maximum, minimum, and mean values of the landscape ecological function in the study area were all smaller than in 2011, while the mean square error increased from 0.176 to 0.194, and the regional differences rose. Among them, only the southeastern part of Hulan District and the northern part of Daowai District improved, most of these areas were the floodplain on both sides of the Songhuajiang.

#### Landscape ecological sustainability

**Figure 7:**
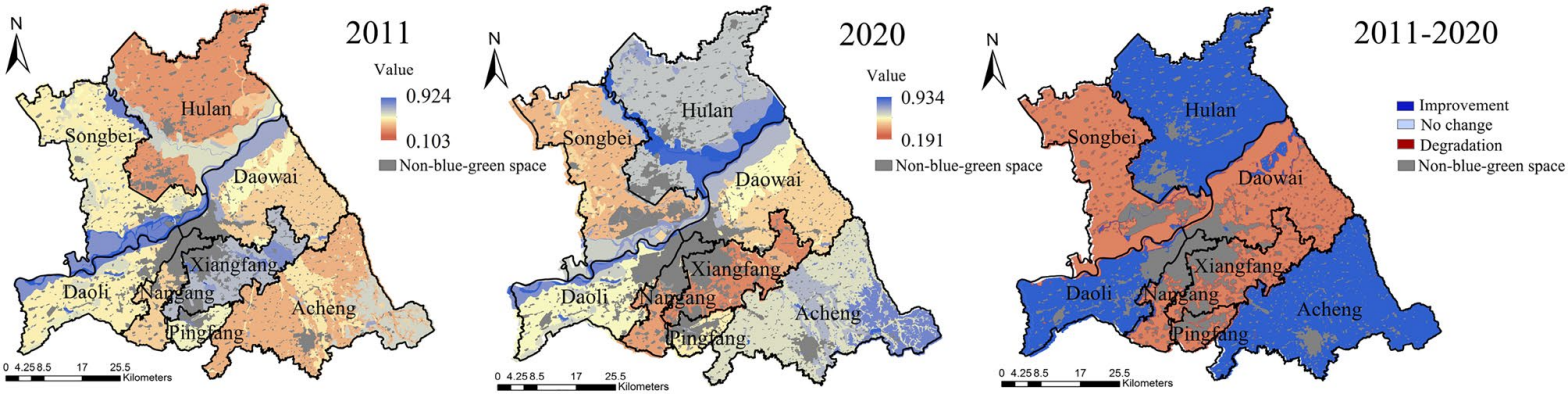
Spatial distribution of landscape ecological sustainability. The Figure is created using ArcGIS ver.10.2 (https://www.esri.com/).

The low values of landscape ecological sustainability in the study area were mainly distributed in Hulan, Acheng, and Nangang Districts, and the high values mainly emerged in Nangang District and along the Songhuajiang in 2011 (Fig. 7). In 2020, the maximum, minimum, and mean values of landscape ecological sustainability increased, and the mean square error decreased from 0.158 to 0.149. The improvement areas were concentrated in Hulan, Daoli, Daowai, Pingfang, and Acheng Districts. The degradation areas were mainly found in Songbei, Daoli, and Nangang Districts. As the Environmental construction input (ECI) of the Harbin government has district difference characteristics, it leads to the change of landscape ecological sustainability also showed district difference characteristics and degradation in Songbei, Nangang, and Xiangfang Districts.

### Assessment of blue–green space LEH

**Figure 8:**
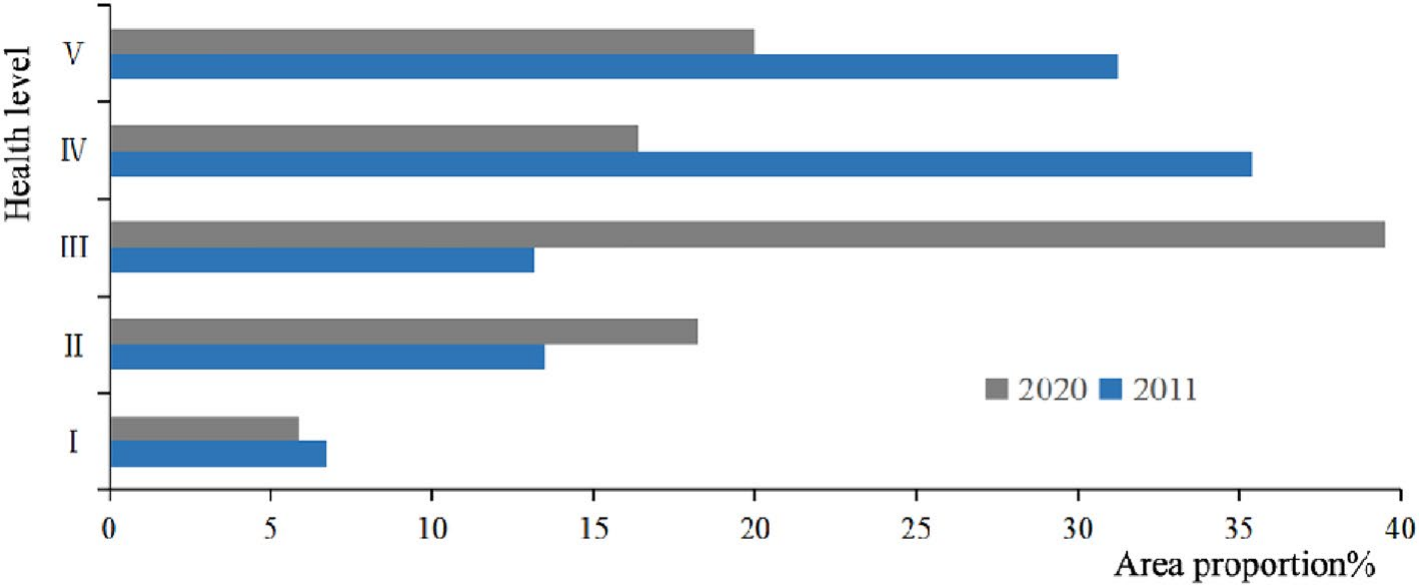
Area proportion of blue–green space LEH level.

**Figure 9:**
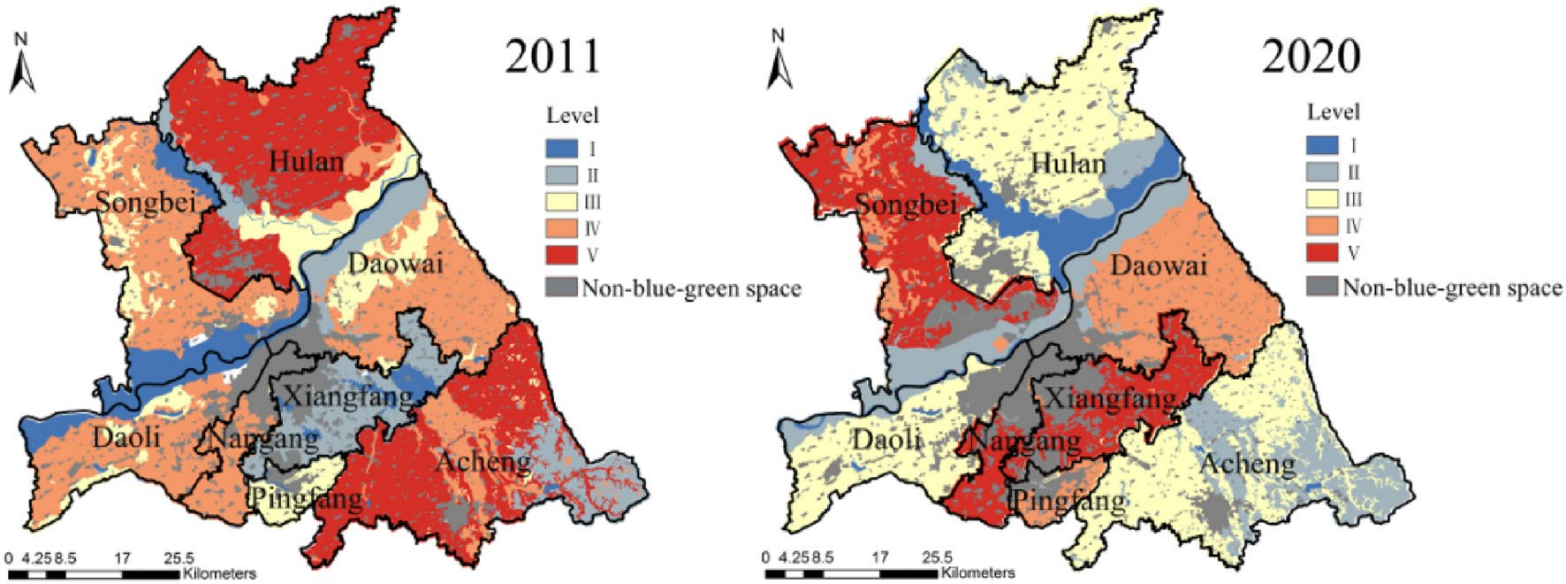
Spatial distribution of blue–green space LEH level. The Figure is created using ArcGIS ver.10.2 (https://www.esri.com/).

The mean value of blue–green space LEH of the study area from 2011 to 2020 increased from 0.377 to 0.386, with slightly improved but still at an unhealthy level, and the mean square error changed from 0.140 to 0.149, with the spatial difference of the overall enhancement. In 2011, the LEH was dominated by the level IV (Fig. 8), the area proportion accounted for 35.392%, which was concentrated in Songbei, Daoli, and Daowai Districts (Fig. 9). Level V had a large-scale distribution in the study area, with an area proportion of 31.248%, focused on Hulan and Acheng Districts. The level III was the most widely distributed in the study area, except for Nangang District. While the level I and the level II mainly emerged in Xiangfang District, the forestland in the east of Acheng District, and the Songhuajiang. In 2020, the dominant level of LEH transferred to level III, and the area proportion raised from 13.162% in 2011 to 39.507% in 2020, mainly distributed in Hulan, Daoli, and Acheng Districts. The area proportion of level IV decreased by 19.992%, with the most noticeable decrease, mainly concentrated in Daowai District, and the area proportion of levels I, II, and V increased in 2020.

#### Assessment of various types blue–green space LEH

**Figure 10:**
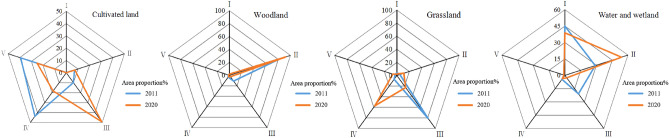
Area proportion of various types blue–green space LEH level.

From 2011 to 2020, among the various types of blue–green space, the most drastic change in LEH level was found in cultivated land (Fig. 10). In 2011, the LEH level was mainly distributed in IV and V. In 2020, the level III became the dominant of cultivated land LEH, with the area proportion increasing from 9.846 to 49.166%. There was an overall trend of improvement in the woodland LEH and was mainly concentrated in level II, with the area proportion increasing from 82.150 to 91.896% during the study period. The grassland LEH level degraded sharply from 2011 to 2020, the area proportion of level III decreased rapidly from 80.843 to 22.772%, and level IV became the main level with an area proportion of 57.114%. As for the LEH level of water and wetland, the area proportion of level I decreased from 44.941 to 38.808% and level II increased from 28.837 to 53.743% during the study period, with a slight decrease in overall LEH.

#### Level transfer of blue–green space LEH

**Figure 11:**
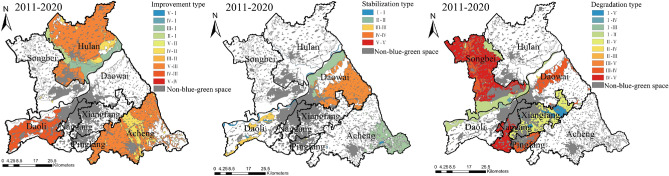
Spatial distribution of blue–green space LEH transfer type. The Figure is created using ArcGIS ver.10.2 (https://www.esri.com/).

From 2011 to 2020, the level transfer type of blue–green space LEH in the study area showed that the improvement type accounted for the largest area proportion, which was 48.297%, mainly distributed in Hulan, Daoli, and Acheng Districts (Fig. 11). Of them, the V-III accounted for the largest area proportion of the improvement type, amounting to 63.572%, mainly distributed in Hulan and Acheng Districts. The IV-III accounted for 13.240% area proportion of the improvement type, which was concentrated in Daoli District with sufficient water resources and relatively flat terrain. The III-II, IV-I, V-I, and V-IV all accounted for a minor area proportion, with a total area of 18.117 km^2^, scattered at the junction of Dawai and Hulan Districts and the northwestern part of Acheng District.

Among the stabilization type, the IV-IV accounted for the largest area proportion, amounting to 48.835%, gathered in the north of Daowai District. The landscape ecological function of these areas balanced the negative impact of pattern, and the values of landscape ecological process and sustainability changed slightly during the study period, leaving the LEH level unchanged. The area proportion of the V-V was the lowest in the stabilization type, mainly distributed at the junction of Hulan and Songbei Districts, and the junction of Acheng and Xiangfang Districts. The value of landscape ecological process in the above areas was relatively low, while the values of pattern, function, and sustainability were comparatively high so that the LEH maintained the same level.

The degradation type was mainly located in Songbei, Nangang, Pingfang, and Xiangfang Districts and the northwestern part of Daowai District, accounting for 33.295% area proportion of the blue–green space. Of which, the IV–V was the largest area proportion, accounting for 40.638% of the degradation type, concentrated in Songbei and Nangang Districts. The transfer types with greater degradation of the I–V, I–IV, and II–V accounted for a total of 19.097% area proportion of the degradation type, with a total area of 220.955 km^2^, mainly emerged in the central and southeastern parts of Acheng District, the northwestern part of Hulan District.

### Spatial autocorrelation analysis

**Figure 12:**
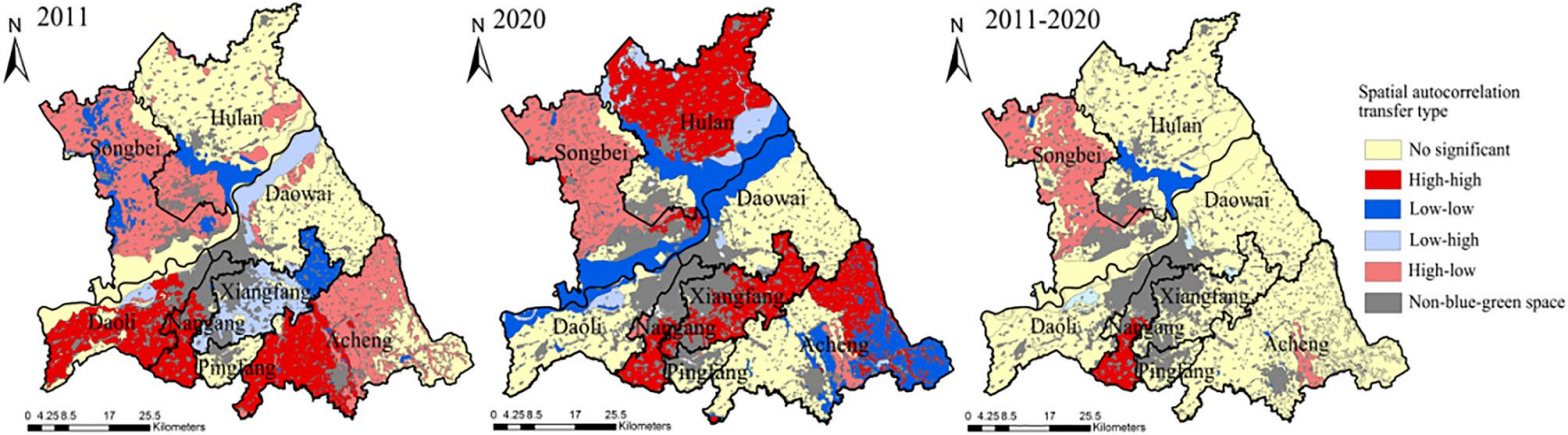
Local spatial autocorrelation types of blue–green space LEH. The Figure is created using ArcGIS ver.10.2 (https://www.esri.com/).

In 2011, the global spatial autocorrelation Moran's I index of blue–green space LEH in the study area was 0.145, with significant positive spatial correlation and strong spatial agglomeration. In 2020, the Moran's I index was increased to 0.148, and the spatial agglomeration increased. From 2011 to 2020, the dominant type of local spatial autocorrelation of blue–green space LEH changed from HL type to HH. In terms of the spatial distribution changes of local spatial autocorrelation types, the HH type was concentrated in the south of the study area in 2011 and mainly distributed in the central part of the study area and Hulan District in 2020. The LL type shifted from the northwestern part of the study area to the Songhuajiang and coastal wetland, and the southeastern part of the Acheng district during the study period. The LH type gradually shifted from the south bank of the Songhuajiang and the cultivated land in Xiangfang District to the north bank of the Songhuajiang from 2011 to 2020. The HL type mainly appeared in Songbei District and Acheng District in 2011 and it was concentrated in the Songbei District in 2020 (Fig. 12).

## Discussion

### Advantages of the assessment framework

Blue–green space is a network system consisting of various ecological spaces^23^. The overall evaluation of various ecological spaces that we conducted could enrich the research scope of LEH and provide more guidance for urban ecological construction. An accurate assessment of LEH is the basis for recognizing regional and stage differences in the evolution of urban ecological health. At present, existing LEH research either overemphasizes the balanced state of the landscape away from disturbance or ignores the effect mechanism of landscape ecology. The majority of research means assessing LEH through the changes in internal landscape pattern or external service function performance^15,38^, leading to large differences in the assessment results. Moreover, landscape ecological sustainability has been considered an important research topic in landscape ecology for more than a decade, but it has not been addressed in existing LEH evaluation studies. Thus, the research results are unable to support the comprehensive and sustainable development of urban ecology. A comprehensive and integrated study of the units involved in LEH is the key to solving the problem.

In this study, based on the analysis of the effect mechanism of urban blue–green space LEH, we constructed an integrated assessment framework of pattern, process, function, and sustainability. The framework not only focused on the real-time health status of the landscape ecosystem but also emphasized the long-term (intergenerational) sustainability of the landscape ecosystem. The assessment system could provide a more scientific and comprehensive integrated framework for understanding the complex interactions between blue–green space and socio-economic systems as well as human-land relationships. It could screen out the key or vulnerable landscape ecosystem pattern characteristics, critical landscape ecological processes, important ecosystem functions, the constraints to sustainable development, and provide specific directions for optimizing the landscape ecosystem. Meanwhile, the proposed assessment framework can contribute to the methodological extension, and could be applied to other rapidly urbanizing regions. In addition, the research results made up for the shortcomings of unilateral analysis in previous studies, which has significant practical implications for achieving the goal of constructing cities with economic prosperity, human health, and ecological harmony worldwide.

### Discussion of assessment units

The degraded areas of landscape ecological pattern in the study area were generally with a single patch type and only a small number of large-scale ecological patches. There are three elements of landscape ecological pattern: patch, corridor, and matrix. In recent years, scholars have combined “organic renewal” and “urban renovation” to optimize the urban ecosystem, that is, by adjusting the number and form of ecological patches, organizing and integrating the ecological patches with the blue channels and green corridors^35,58^. The practices confirm that enhancing the connectivity of landscape patches is the most effective way to optimize landscape ecological pattern.

The landscape ecological process is the key optimization unit of blue–green space LEH according to the current status in the study area and its assessment weights. From 2011 to 2020, the degraded area of landscape ecological process was highly coincident with the spatial expansion scope of “leap north and west, extend south and east, and expand in the middle and outside” proposed by the Harbin municipal government in 2011. Urban construction activities changed the blue–green space from dense clusters to dispersed clusters^26,39^, which brought a series of negative impacts on landscape ecological processes. Such as reduced ecological stability, less internal biodiversity, and blocked material and energy transfer. There are numerous studies that also confirm that urban construction activities are the main factor affecting landscape ecological efficiency^5,9^.

From 2011 to 2020, the landscape ecological function decreased in most parts of the study area, except for the floodplain on both sides of the Songhuajiang. Due to long-term river flood erosion and river sand accumulation, has formed a large area of wetland landscape on both sides of the Songhuajiang. The water system in the area is rich and self-contained, and the landscape ecological function has been improved, the assessment result was consistent with the findings of other scholars^22,39^. The degradation of landscape ecological function will destroy the balanced land-use structure. Meanwhile, the land-use structure will have an impact on the formation, supply, and distribution of landscape ecological function, which form a mutual restriction and promotion relationship^43,45,55^.

Landscape ecological sustainability of the study area was greatly influenced by ECI, showing obvious district differences, which were related to the ecological construction priorities of the districts. Among them, Hulan and Acheng Districts had larger ecological construction input, mainly for environmental pollution prevention and control. Daoli and Daowai Districts were mainly used for the renovation of farmland ecosystem in the urban fringe area. And the Pingfang District was mostly for the treatment of heavy industry and heavy pollution. While other districts were mainly spent on urban infrastructure improvement. Therefore, landscape ecological sustainability requires scientific adjustment of environmental construction input, and obtaining the maximum ecological output with the least input is a matter of considerable concern.

In the study area, the spatial changes of the four assessment units of LEH: pattern, process, function, and sustainability were not coordinated. The assessment results further proved that there are limitations in assessing LEH by relying only on landscape pattern or external functional performance. Optimization of urban blue–green space LEH should be to promote the unity, coordination of pattern, process, function, and sustainability based on strengthening the weak units in the landscape scale. According to the results of the study, increasing the connectivity of ecological patches is the key to improving the landscape ecological pattern. The landscape ecological process is subject to the expansion of non-ecological land. A comprehensive adjustment of land-use structure is an effective way to improve landscape ecological function. And the landscape ecological sustainability is closely related to the scientific input of ecological construction. Discussing the change characteristics of landscape ecological pattern, process, function, and sustainability in the study area could be a targeted reference for LEH optimization in Northeast China and other cities with the same type of development.

### Discussion of LEH assessment results

From 2011 to 2020, the blue–green space LEH in the study area was improved, and the major level was transformed from IV to III, but the spatial difference increased, and this assessment result is consistent with the previous studies^20,22,24^. It can be seen that since the introduction of the “12th Five-Year Plan for the Revitalization of Northeast China”, Northeast China has actively practiced the concept of green development and ecological construction and achieved certain results. Yet, we also found that the LEH of blue–green space in the study area was still at an unhealthy level, there was a large space to be improved.

From 2011 to 2020, the improvement type of LEH accounted for the largest area proportion. These areas were generally distributed in the ecological input priority areas, how to get rid of excessive reliance on financial investment, and use the effect mechanism of LEH to optimize the blue–green space is urgent. The IV–V had the largest area proportion of the degradation type, indicating that areas with a worse LEH foundation are more likely to be degraded. The I–V, I–IV, and II–V degradation types with large level decline accounted for a small area proportion, but will have a strong negative impact on the overall LEH of the study area and should be given full attention.

As for the changes in various types of blue–green space LEH in the study area, the most significant LEH degradation occurred in grassland. The same results were also found in related studies of other cities with rapid urbanization^16,17^. Urban grassland mostly comes from urban settlements and parks, and the development of urbanization has made it increasingly artificial. This will certainly lead to the decline of structural and functional integrity^50^, and eventually the degradation of grassland LEH. The improvement of the woodland LEH in the study area was mainly due to the implementation of the National Ecological Environment Construction Plan during the study period. Urban woodland is generally less disturbed in general, but once destroyed, later ecological recovery is slow. Therefore, attention must be paid to the woodland protection to prevent LEH degradation^21,26^. The improvement of cultivated land LEH in the study area was directly related to the optimal deployment of agricultural cropping structures and the construction of high-standard basic farmland^23^. Such a series of cultivated land adjustment measures are of great value to other regions. The decline of water and wetland LEH in the study area was mainly due to the support policies for agriculture development. Excessive exploitation of groundwater for cultivated land irrigation has led to a serious decline in the groundwater level and an imbalance in the dynamic balance of water resources, leaving its LEH at risk. From the above analysis, we can conclude that optimizing the internal pattern of grassland, avoiding woodland from external interference, implementing effective cultivated land protection measures, scientifically adjusting the land-use structure of blue–green space, and developing high standard watershed planning and management methods are essential for the LEH of blue–green space.

### Implications of spatial autocorrelation analysis

The relevant theories and practices of regional development show that there is a diffusion or polarization effect between regions, which can reduce or enlarge regional spatial differences. HH and LL types of local spatial autocorrelation can be regarded as an objective reflection of the spatial diffusion effect. While HL and LH types are a manifestation of the spatial polarization effect^18^, this effect will show an intensified trend under the “Matthew effect” of spatial correlation. For this reason, it is necessary to clarify the effect characteristics of local spatial autocorrelation types, which will be a new perspective to explore urban blue–green space LEH optimization methods using spatial correlation effects.

The HH type is a high aggregation area of blue–green space LEH high levels, where has an obvious diffusion effect in the region and has a positive contribution to the overall blue–green space LEH in the region^21^. As these blue–green spaces have high levels of LEH, it should continue to strengthen protection measures and enhance the impact of diffusion effects with these areas as the center in the future.

The LL type is a high aggregation area of blue–green space LEH low levels, where has an obvious diffusion effect in the region and is the primary area for LEH restoration. For such areas, their improvement should be treated differently. It is necessary to actively promote various landscape ecological improvement means and protection implementation from an integrated perspective. In addition, if there is a land requirement for conversion of blue–green space to non- blue–green space, the area where LL type is located is the relatively ideal choice^21,58^.

The LH type is similar to a concave, that is, the blue–green space with LEH high levels is distributed around the low levels^15,21^. Under the influence of the spatial polarization (echo) effect, the areas with LEH low levels are easily assimilated by the surrounding areas with LEH high levels and then evolve into the HH type. For such areas, we should focus on curbing the effects of polarization in areas with LEH low levels, actively cultivating the natural conditions of blue–green space with LEH low levels. And making full use of the diffusion effect of surrounding areas with high LEH levels to accelerate the transition to HH type.

The HL type is similar to a convex, that is, the blue–green space with LEH high levels is concentrated in the middle, surrounded mostly by LEH low levels. Under the influence of the spatial polarization (echo) effect, the areas with LEH high levels are easily assimilated by the surrounding areas with LEH low levels and then evolve into the LL type^21,23^. For such areas, we should actively protect the areas with LEH high levels that produce polarization effects. It is necessary to isolate or reduce the influence of the surrounding areas with LEH low levels on them, and gradually expand the protection scope to promote the overall improvement of LEH.

### Limitations and prospects

Some uncertainties and limitations also exist in this study, there may be uncertainties in the calculation of metrics due to inconsistent spatial resolution of data or excessive parameter settings.

Meanwhile, it failed to clarify the driving mechanism of blue–green space LEH and the effect of its optimization on the future development of the city. Thus, in the subsequent research, we will explore the effects of multiple factors on blue–green space LEH and conduct multiple scenarios simulation. So as to reduce the problems caused by uncertainty factors, maintaining the sustainable development capacity of blue–green space, and identifying more effective measures to optimize the LEH of blue–green space.

## Conclusions

In this study, by clarifying the effect mechanism, we constructed an urban blue–green space LEH assessment framework based on the integration of pattern, process, function and sustainability. The assessment framework can contribute to the methodological extension, and could be applied to other rapidly urbanizing regions. The results showed that the spatial changes in the four assessment units of landscape ecological pattern, process, function and sustainability were not coordinated in the study area. The overall condition of blue–green space LEH improved but still at an unhealthy level, and the spatial difference increased during the period of 2011 to 2020 in the study area. Grassland, water and wetland in the study area suffered from the widespread degradation of LEH, while cultivated land and woodland showed an overall trend of improvement in the LEH. The LEH level improvement type had the largest area proportion, and the stabilization type had the smallest. The spatial agglomeration of blue–green space LEH in the study area increased, and its local spatial autocorrelation dominant type changed from HL to HH, with large differences in the spatial distribution in 2011 and 2020. These findings are vital for promoting the creation of a more harmonious and sustainable human living environment, and can provide significant references for urban blue–green space protection and management.
